# Non-Linear Pressure Sensitivity of Standard Telecommunication Cables

**DOI:** 10.3390/s26113618

**Published:** 2026-06-05

**Authors:** Abdulfatah A. G. Abushagur, Mohd Ridzuan Mokhtar, Noor Shafikah Md Rodzi, Siti Azlida Ibrahim, Khazaimatol Shima Subari, Zulkifli Mahmud, Hairul Azhar Abdul Rashid, Andre Franzen, Zulfadzli Yusoff

**Affiliations:** 1 Centre of Fibre Networking and Communication, COE for Intelligent Network, Multimedia University, Cyberjaya 63100, Selangor, Malaysia; a.abushagur@mmu.edu.my (A.A.G.A.); azlida@mmu.edu.my (S.A.I.); 1171402881@student.mmu.edu.my (Z.M.); hairul@mmu.edu.my (H.A.A.R.); zulfadzli.yusoff@mmu.edu.my (Z.Y.); 2 Faculty of Artificial Intelligence and Engineering, Multimedia University, Cyberjaya 63100, Selangor, Malaysia; khazaimatol@dkss.com.my; 3 Petronas Research Centre SDN BHD, Kawasan Institusi Bangi, Jalan Ayer Hitam, Kajang 43000, Selangor, Malaysia; andref@franzen-my.com

**Keywords:** distributed pressure sensing, opportunistic sensing, strain transfer, telecommunication cables, TW-COTDR

## Abstract

The utilization of existing telecommunication infrastructure for environmental monitoring via opportunistic sensing is rapidly advancing the field of distributed fiber optic sensing (DFOS). However, while custom-engineered sensing cables are highly characterized for hydrostatic pressure, the complex mechanical response of standard armored telecommunication networks remains largely unquantified. This study experimentally investigates the non-linear distributed pressure sensitivity of three commercial telecommunication cables (Anti-Rodent, Duct, and Microcable) across a hydrostatic pressure range of 0 to 800 PSI. Measurements were conducted using Tunable Wavelength Coherent Optical Time Domain Reflectometry (TW-COTDR) with a 20 cm spatial resolution, utilizing a stepped depressurization protocol with 15-min stabilization holds to isolate true steady-state longitudinal strain. The results reveal that protective cable armoring induces severe mechanical non-linearity. The rigid Glass Reinforced Plastic (GRP) rods of the Anti-Rodent cable acted as a structural vault at low pressures before yielding to become highly sensitive above 400 PSI. Conversely, the corrugated steel tape of the Duct cable exhibited high initial sensitivity followed by mechanical stiffening, while the unarmored Microcable maintained a linear response. These findings establish that a single linear calibration coefficient is invalid for heavily armored infrastructure, highlighting the critical need for structural characterization prior to opportunistic field deployments.

## 1. Introduction

Distributed fiber optic sensing (DFOS) is rapidly transforming environmental monitoring and critical infrastructure management by offering continuous, long-range measurement capabilities. A major driving force in this field is “opportunistic sensing” leveraging existing telecommunication networks and “dark fiber” to monitor environmental and seismic activity without the cost of laying new cables [1,2]. Within this scope, recent advancements have successfully demonstrated the use of DFOS in large-scale water infrastructure, such as applying fiber optics to High-Density Polyethylene (HDPE) pipelines to detect pressure anomalies and water leakage [3]. Furthermore, the demand for high-spatial-resolution distributed pressure sensors is growing in dynamic environments, particularly for tracking propagating solitary waves in coastal hazard monitoring and tsunami early warning systems [4].

Despite these advantages, distributed hydrostatic pressure sensing remains a significant challenge. Rayleigh-based distributed sensing allows continuous spatial interrogation of longitudinal strain along a fiber [5,6]; however, bare silica optical fibers possess a high Young’s modulus and are largely insensitive to uniform hydrostatic pressure. To achieve reliable pressure measurements, researchers typically rely on custom-engineered sensing cables where the geometry is heavily modified to convert external pressure into longitudinal stretch and compression along the fiber [6,7,8,9]. For example, highly sensitive sensors have been developed by winding an optical fiber helically around a highly compressible polymer core, which effectively transduces external radial pressure into measurable axial strain [10]. These specialized, custom-built designs have proven highly successful when embedded in geohydraulic structures to capture pore water pressure variations in real-time [11].

However, utilizing standard telecommunication infrastructure for opportunistic pressure sensing introduces a critical mechanical divergence. While engineered sensors utilize soft, compressible materials to yield to external pressure, standard telecommunication cables are explicitly designed with rigid armoring (such as Glass Reinforced Plastic rods or corrugated steel tape) to isolate internal fibers from environmental forces and prevent crushing. Previous studies have shown that cable structure, particularly loose-tube designs and protective outer sheaths, significantly dampens the strain transfer coefficient from the external environment to the internal core, often resulting in complex mechanical hysteresis under physical loading [12].

While extensive research has successfully characterized the strain-transfer mechanics of custom-built, highly sensitive cables, the non-linear pressure response of standard armored telecommunication infrastructure remains largely unquantified. Our previous work [13] established the baseline dynamic responses of three such commercial cables (Anti-Rodent, Duct, and Microcable) to acoustic and vibrational excitation using Distributed Acoustic Sensing (DAS). However, that investigation was strictly limited to dynamic vibration-to-strain transfer, measuring relative acoustic amplitudes.

To address this gap and investigate a fundamentally different mechanical and optical regime, the present study characterizes the quasi-static optical response of these same three standard cables (obtained from Dura-Mine Sdn. Bhd. [14]) to sustained hydrostatic loading. Measurements were carried out using a Tunable Wavelength Coherent Optical Time Domain Reflectometry (TW-COTDR) interrogator (Neubrex Co., Ltd., NBX 7000 series [15]) to measure the absolute Rayleigh frequency shift (GHz) as a direct function of pressure. By shifting from transient acoustic excitation to steady-state pressure-to-longitudinal-strain transfer, this study provides the quantitative framework necessary to identify the absolute yielding thresholds and non-linear compressive locking behaviors of the structural armoring phenomena that cannot be captured under dynamic acoustic loading. Ultimately, this work provides a novel experimental demonstration of these complex strain-transfer behaviors, proving that standard commercial armoring undergoes non-linear structural yielding that dictates an entirely new calibration context for future opportunistic distributed pressure deployments.

## 2. Experimental Setup and Methodology

### 2.1. Cable Samples and Interrogation System

**Figure 1:**
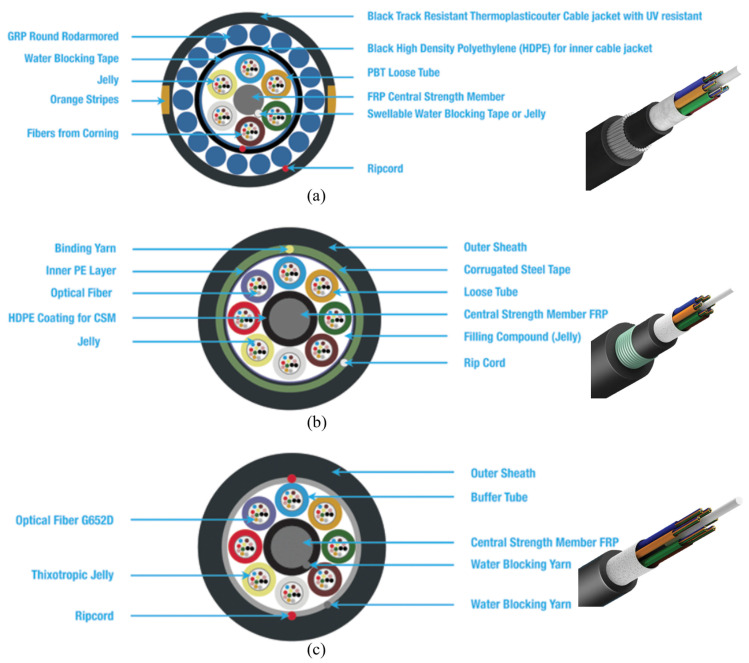
Cross-sectional structures of the three commercial telecommunication cables evaluated for distributed pressure sensing: (**a**) Anti-Rodent cable featuring rigid Glass Reinforced Plastic (GRP) rods. (**b**) Duct cable utilizing corrugated steel tape armor. (**c**) Lightweight Microcable relying on a thin outer sheath.

As introduced, the three selected telecommunication cables represent the most common structural deployments in opportunistic sensing environments (Figure 1). To evaluate the impact of their standard armoring on distributed pressure sensitivity, a single continuous length of each cable type was selected. Each cable relies on a distinct mechanical barrier: (a) an Anti-Rodent cable featuring rigid Glass Reinforced Plastic (GRP) rods, (b) a Duct cable utilizing corrugated steel tape, and (c) a lightweight Microcable relying primarily on a thin outer sheath.

To contextualize the distinct mechanical yielding behaviors observed under hydrostatic load, the specific structural dimensions and materials of these test samples are detailed in Table 1.

For optical interrogation, the TW-COTDR system (Neubrex NBX-7000, Neubrex Co., Ltd., Sakaemachidori, Osaka, Japan) was configured with a spatial resolution of 20 cm, a frequency sweep range of 300 GHz, and a frequency step resolution of 250 MHz to continuously track the Rayleigh frequency shift along the fiber and determine relative changes in longitudinal strain.

### 2.2. Pressure Chamber Configuration

**Figure 2:**
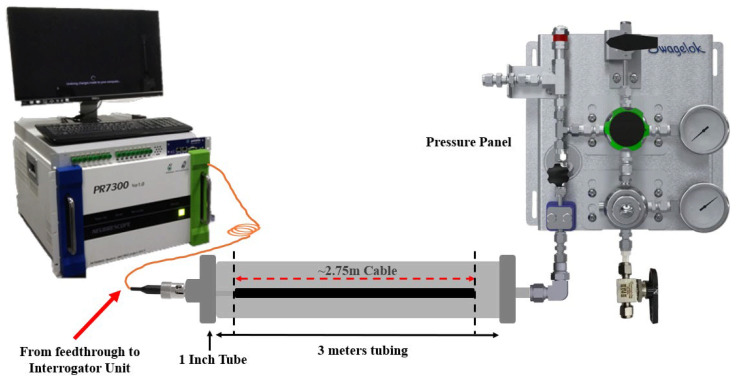
Schematic of the hydrostatic pressure testing facility. A 2.75 m length of each cable sample was housed within a water-filled, 3-m rigid steel chamber and monitored via a TW-COTDR interrogator to capture continuous Rayleigh frequency shifts under controlled pressurization.

A specialized hydrostatic pressure testing facility was constructed to isolate and control the pressure response of the cables. Approximately 2.75 m of each fiber cable sample was inserted into a 3-m-long, 1-inch-diameter rigid tube chamber (Figure 2). To serve as an efficient hydrostatic medium and significantly accelerate the pressurization process, the chamber was filled with water to 80–90% capacity before sealing.

Following this, pressure gradually applied to the chamber until it reached full capacity. During this process, the release valve was kept open to allow trapped air to escape while ensuring that the water remained inside the chamber. Once the chamber was completely filled and all trapped air had been expelled, the release valve was closed. This helped maintain a stable pressure condition without interference from residual air. After the chamber was properly filled and sealed, the internal optical fibers were routed through a high-pressure feedthrough at one end of the chamber to connect to the interrogator, while the opposite end was connected to a controlled pressure panel. Internal pressure was regulated via a hydraulic hand pump and monitored by a reference digital gauge with an accuracy of ±0.5% full scale.

### 2.3. Stepped Pressure Protocol

Because standard telecommunication cables contain gel-filled loose tubes and complex protective layers, the strain transfer from the external environment to the internal fiber is subject to mechanical hysteresis and delayed relaxation. To ensure steady-state measurements, a stepped depressurization protocol was implemented. The chamber was initially pressurized to a maximum of approximately 800 PSI. Data recording was deliberately conducted from high pressure to low pressure, as this descending directional approach allowed for more stable and precise control of the testing environment.

**Figure 3:**
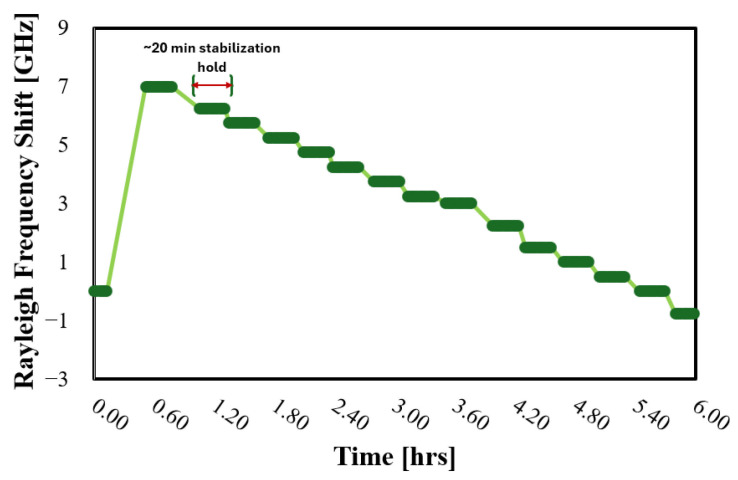
The step depressurization protocol utilized for mechanical stabilization. To isolate true steady-state strain transfer from transient mechanical hysteresis, hydrostatic pressure was released from ~800 PSI in decrements of ~50 PSI, with strict 15-min stabilization holds applied at each step.

The pressure was systematically released in decrements of approximately 50 PSI. Crucially, each specific pressure magnitude was held constant for ~15–20 min (Figure 3).

This stabilization hold allowed the cable structures to mechanically equilibrate, ensuring that the accompanying Rayleigh frequency shift observed by the interrogator represented the true steady-state strain transfer rather than a transient mechanical response.

Furthermore, filling the chamber to full capacity with water provided a significant thermal mass, minimizing adiabatic temperature fluctuations during pressure transitions. The ~20-min stabilization holds at each 50 PSI decrement ensured that the internal testing environment maintained strict thermal equilibrium with the constant ambient laboratory temperature, thereby eliminating temperature cross-sensitivity from the recorded Rayleigh frequency shifts.

The hydrostatic pressure range of 0 to 800 PSI (approximately 0 to 5.5 MPa) was selected to model the extreme ambient conditions of subsea opportunistic sensing environments, corresponding to roughly 550 m of water depth. Furthermore, this elevated range was a strict methodological requirement to fully characterize the mechanical strain-transfer envelope of the cables’ heavy structural armoring. Testing up to 800 PSI ensured that any latent non-linear mechanical transitions within these components would be successfully captured.

## 3. Results and Discussions

### 3.1. Non-Linear Pressure Regimes in Standard Cables

**Figure 4:**
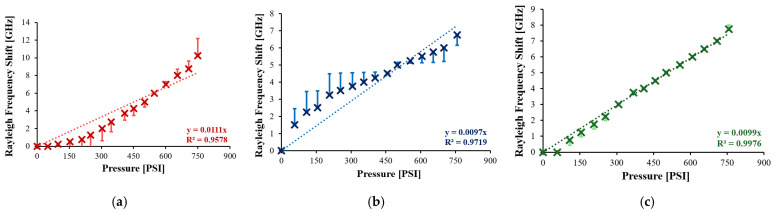
Rayleigh frequency shift as a function of hydrostatic pressure for the three standard telecommunication cables: (**a**) Anti-Rodent cable, (**b**) Duct cable, and (**c**) Microcable. The dotted lines represent the theoretical linear calibration fits. Error bars indicate the absolute deviation (residual) of the measured TW-COTDR data from the linear prediction, highlighting the failure of the linear assumption at elevated pressures for the armored variants.

The steady-state Rayleigh frequency shift for the three standard telecommunication cables was recorded across a hydrostatic pressure range of 0 to 800 PSI. As illustrated in Figure 4, plotting the frequency shift against applied pressure reveals that heavy cable armoring fundamentally alters the strain transfer mechanics, preventing a simple linear calibration. To quantify this non-linear behavior, a bi-linear sensitivity analysis was conducted, dividing the pressure response into a low-pressure regime (0–300 PSI) and a high-pressure regime (400–800 PSI), summarized in Table 2.

### 3.2. Baseline Strain Transfer: The Microcable

The Microcable served as a structural baseline due to its lack of heavy metallic or rigid composite armoring. Because external pressure only had to overcome a relatively thin outer sheath and water-blocking yarn, the cable exhibited a consistent and predictable strain transfer. The sensitivity remained largely linear across the entire testing range, shifting only slightly from 0.0089 GHz/PSI in the low-pressure regime to 0.0100 GHz/PSI at elevated pressures (see Figure 4c for the absolute residual variance at each pressure interval).

### 3.3. Armor Yielding: The Anti-Rodent GRP Cable

The most severe non-linear behavior was observed in the Anti-Rodent cable. In the low-pressure regime, the rigid ring of Glass Reinforced Plastic (GRP) rods acted as a highly effective structural vault, actively resisting radial compression and isolating the internal fibers. This resulted in an exceptionally low sensitivity of ~0.0050 GHz/PSI. However, as hydrostatic pressure exceeded 400 PSI, the GRP armor structure yielded. Once this mechanical threshold was breached, radial pressure was rapidly transferred to the core, causing the sensitivity to surge by over 270% to 0.0186 GHz/PSI (see Figure 4a for the absolute residual variance at each pressure interval). Consequently, at high pressures, this heavily armored cable paradoxically became the most sensitive of the three samples.

### 3.4. Armor Stiffening: The Duct Cable

Conversely, the Duct cable, protected by corrugated steel tape, demonstrated a compressive “stiffening” effect. In the low-pressure regime, the corrugated structure compressed relatively easily, yielding a high initial sensitivity of 0.0143 GHz/PSI. As the pressure increased beyond 300 PSI, the corrugations fully compressed, causing the steel tape to stiffen and effectively resist further longitudinal strain transfer. This mechanical bottoming-out reduced the high-pressure sensitivity by nearly 35% to 0.0092 GHz/PSI (see Figure 4b for the absolute residual variance at each pressure interval).

**Table 2:** Bi-linear distributed pressure sensitivity (DPS) coefficients for the tested cables, demonstrating structural yielding and stiffening across low-pressure (0–300 PSI) and high-pressure (400–800 PSI) regimes.

Cable Type	Low-Pressure Sensitivity (0–300 PSI)	High-Pressure Sensitivity (400–800 PSI)
Microcable	0.0089 GHz/PSI	0.01 GHz/PSI
Duct Steel Tape	0.0143 GHz/PSI	0.0092 GHz/PSI
Anti-Rodent GRP	0.005 GHz/PSI (Armor resists)	0.0186 GHz/PSI

### 3.5. Limitations and Deployment Relevance

These results demonstrate that the mechanical structures engineered to protect standard telecommunication cables introduce significant non-linearity into distributed pressure measurements. The assumption of a single, static calibration coefficient often utilized in custom-engineered sensing cables is invalid for opportunistic deployments. Depending on the armor type, a cable may act as an isolator at low pressures and a highly sensitive transducer at high pressures, or vice versa.

While the controlled hydrostatic testing in this study successfully isolates these fundamental yielding behaviors, it is necessary to distinguish these steady-state laboratory characterizations from actual opportunistic field deployments. Specifically, while real-world dynamic sensing events typically produce minor pressure variations (e.g., 1–5 PSI), the expansive 0–800 PSI testing range utilized here is required to map the broad envelope of static baseline deployment depths upon which those small dynamic fluctuations are superimposed. Furthermore, under actual operating conditions, distributed sensing cables are subjected to complex, multi-axial strain profiles rather than uniform hydrostatic pressure alone. Factors such as localized compression, bending, soil friction in direct-buried applications, and residual installation stresses will superimpose additional variables onto the optical phase response. As this study serves as an initial phenomenological baseline using single-sample profiles, future statistical investigations will be necessary to quantify specific batch-to-batch manufacturing tolerances of these commercial cables.

Consequently, the practical calibration strategy for opportunistic sensing networks cannot rely on averaged coefficients for a given cable class. Because installation mechanics and localized boundary conditions will inherently shift the specific yielding thresholds of the armoring, individual, in situ calibration of each deployed line is strictly required. Practical field calibration must be conducted against localized reference sensors (e.g., adjacent pressure gauges at subsea nodes or wellheads) during system commissioning to map the exact transition regimes of the installed infrastructure. Finally, because the long-term stability of these thresholds under repeated dynamic loading, hysteresis, and material aging remains an open parameter, future longitudinal field studies will be essential to establish comprehensive lifecycle calibration models.

## 4. Conclusions

This study experimentally characterized the distributed pressure sensitivity (DPS) of three standard commercial telecommunication cables (Microcable, Duct, and Anti-Rodent) to evaluate their mechanics for opportunistic environmental monitoring. Utilizing a stepped depressurization methodology and TW-COTDR interrogation to isolate steady-state longitudinal strain, the results definitively show that protective cable armoring induces highly non-linear strain-transfer regimes. Specifically, the rigid GRP rods of the Anti-Rodent cable created a structural vault effect at low pressures before yielding to become highly sensitive above 400 PSI. Conversely, the corrugated steel tape of the Duct cable exhibited high initial sensitivity but mechanically stiffened at elevated pressures, while the unarmored Microcable maintained a relatively linear response.

Based on these characterized mechanical thresholds, distinct applicability ranges can be established for future opportunistic deployments. The Microcable, which exhibited highly linear strain-transfer across the entire 0–800 PSI range, is the most predictable candidate for broad-spectrum, continuous pressure monitoring, provided the deployment environment does not strictly dictate heavy mechanical protection. Conversely, the corrugated steel tape of the Duct cable demonstrates excellent sensitivity at lower pressures before experiencing compressive locking; therefore, it is best suited for shallow-water or low-pressure terrestrial deployments where high-resolution monitoring is required, but extreme hydrostatic crushing is unlikely. Finally, the rigid GRP rods of the Anti-Rodent cable act as a mechanical isolator at low pressures but exhibit a sharp, non-linear yielding transition at high pressures. Consequently, this architecture is optimal for high-stress threshold detection—such as subsea geohazard monitoring or structural failure alerts—where capturing sudden, extreme pressure variations is prioritized over low-pressure precision.

These findings establish a critical limitation for the field: standard telecommunication cables cannot be accurately calibrated for distributed pressure using a single linear coefficient. To ensure reliable data interpretation in opportunistic sensing deployments, the specific yielding thresholds and mechanical behaviors of the existing cable infrastructure must be structurally characterized prior to field implementation.

## Figures and Tables

**Table 1:** Condensed structural specifications of the evaluated commercial optical cables.

Cable Classification	Outer Diameter (mm)	Primary Structural Armoring	Outer Jacket Material
Microcable	~9	GFRP Central Strength Member	Polyethylene (PE)
Duct Cable	~15	Corrugated Coated Steel Tape	Double Sheath Polyethylene (PE)
Anti-Rodent	~19	Rigid Round GRP Rods (1.2 mm)	Double Sheath (HDPE Inner, PE Outer)

## Data Availability

The original contributions presented in this study are included in the article. Further inquiries can be directed to the corresponding authors.
